# “Keeping us on our toes”: a review of what clinicians need to know about vancomycin-variable *Enterococcus*

**DOI:** 10.1017/ash.2024.449

**Published:** 2024-11-11

**Authors:** Marten R. Hawkins, Natalia Medvedeva, Hannah Wang, Niaz Banaei, Marisa K. Holubar

**Affiliations:** 1 Division of Infectious Diseases and Geographic Medicine, Department of Medicine, Stanford University School of Medicine, Stanford, CA, USA; 2 Department of Pathology and Laboratory Medicine, Cleveland Clinic, Cleveland, OH, USA; 3 Department of Pathology, Stanford University School of Medicine, Stanford, CA, USA

## Abstract

*Enterococcus faecium* is a difficult-to-treat gram positive organism with increasing rates of resistance to vancomycin which is commonly mediated through the *vanA* gene cluster. There have been international reports of *E. faecium* isolates that are genotypically positive for *vanA* but phenotypically vancomycin-susceptible. These isolates, commonly called vancomycin-variable enterococci (VVE), can convert to phenotypic vancomycin resistance upon exposure to vancomycin. Multiple mechanisms for this genotypic-phenotypic mismatch have been reported and most commonly involve the regulatory components of the *vanA* gene cluster. VVE are challenging to identify unless microbiology labs routinely implement both genotypic and phenotypic screening methods. VVE has been associated with outbreaks and has become a prevalent pathogen in several countries. In this review, we summarize the mechanisms, microbiology and epidemiology of VVE. Clinicians must remain vigilant for VVE as diagnosis can be challenging and treatment failure on vancomycin is possible.

## Case report

A 62-year-old male with non-ischemic cardiomyopathy requiring a left-ventricular assist device (LVAD) was hospitalized with LVAD driveline drainage. Computed tomography of the chest showed a phlegmon along the LVAD, including the outflow tract, pump, and driveline. On hospital day 6, he underwent surgical incision and drainage. Intra-operative cultures grew a vancomycin-susceptible (MIC 0.5 ug/mL by VITEK) *Enterococcus faecium* isolate. Intravenous vancomycin was started after debridement. On hospital day 15, he underwent repeat surgical incision and debridement with tissue flap reconstruction to achieve closure of his surgical wound. Additional intra-operative cultures obtained during this surgery grew *E. faecium* now with phenotypic vancomycin resistance (MIC >16 ug/mL by VITEK) with an otherwise identical susceptibility profile and detection of the *vanA* gene via molecular testing. *VanA* testing was not performed on the intra-operative swab specimen submitted from the first surgery because it did not meet laboratory criteria for testing, which in our center is isolation from blood or a sterile tissue/fluid specimen. Subsequent molecular testing of the initial phenotypically vancomycin-susceptible *E. faecium* isolate also revealed the presence of *vanA*, consistent with infection due to a vancomycin-variable enterococcal (VVE) isolate.

## Introduction to vancomycin-variable enterococci (VVE)

Although vancomycin-variable enterococci lack a formal definition, the term is typically used to describe enterococcal isolates that harbor the *vanA* gene cluster but remain phenotypically susceptible to vancomycin. The *vanA* gene cluster is the most common mechanism for vancomycin resistance in enterococci. VVE occur when mutations in the *vanA* gene cluster result in a genotypic-phenotypic mismatch. Although more common in *E. faecium, vanA* can rarely be present in *E. faecalis*, and there are some reports of a similar genotypic-phenotypic mismatch.^1^ Because the majority of VVE cases described in the literature to date are *E. faecium*, our review will focus on this species.

## Mechanisms of vancomycin resistance

Vancomycin resistance is common among *E. faecium* isolates worldwide.^2^ At least nine different mechanisms of vancomycin resistance have been described in enterococci: *vanA, vanB, vanC, vanD, vanE, vanG, vanL, vanM,* and *vanN*.^3^ Acquired vancomycin resistance is most commonly mediated by the *vanA* gene cluster through alteration of the glycopeptide binding site. The *vanA* gene cluster is found on transposon Tn1546 which is commonly incorporated into plasmids allowing for transfer and spread across strains and even species.^4^ Other gene clusters (ie, *vanB)* similarly confer vancomycin resistance to enterococci and are named for the enzyme that ultimately modifies the glycopeptide binding site. These other gene clusters generally differ in the enzymes expressed by the operon, the modified terminal sequence at the glycopeptide binding site (eg, D-ala-D-lac vs. D-ala-D-ser), location on genetic elements, type of expression (inducible or constitutive), and degree of resistance conferred to different glycopeptides. The *vanB* gene cluster, for example, can confer resistance to vancomycin while retaining susceptibility to another glycopeptide, teicoplanin. The *vanC* gene cluster, found on chromosomes of *E gallinarum* and *E. casseliflavus-E. flavescens,* confers intrinsic low-level vancomycin resistance while retaining teicoplanin susceptibility.^5^ Although other gene clusters have been described, they are much less common than *vanA, vanB and vanC.* Among *E faecium* isolates, *vanA* remains the most common mechanism conferring vancomycin resistance.

**Figure 1.**
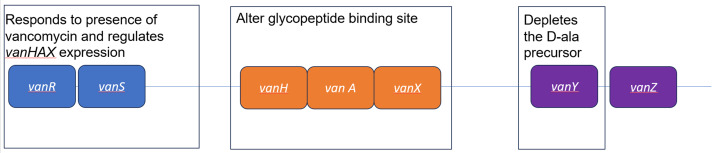
Components of the *vanA* gene cluster and their function. Adapted from Faron et al.^30^

The *vanA* cluster contains several genes: *van R, S, H, A, X, Y,* and *Z* (Figure 1). The proteins encoded by these genes work together to change the glycopeptide binding site’s peptidoglycan terminal sequence (D-ala-D-ala) by replacing the terminal D-alanine with D-lactate, forming D-ala-D-lac. This alters vancomycin’s target site, inhibiting its mechanism of action. Each gene in the *vanA* cluster serves a different function. The protein complex VanR/S includes the regulatory (VanR) and sensory (VanS) components for the rest of the operon. VanR/S regulates *vanHAX* expression by forming a two-component signal transduction system. When VanS detects the presence of glycopeptides, it phosphorylates VanR to promote transcription of *vanHAX*. Enzymes encoded by *vanHAX* specifically alter the glycopeptide binding site. VanX cleaves the peptidoglycan terminal D-ala-D-ala, VanH is a dehydrogenase that produces lactate (D-lac), and VanA is a ligase that forms the bond between D-ala and D-lac. Although *vanA, R, H,* and *X* are considered essential for vancomycin resistance, *vanY* and *vanZ* have a less clear role. VanY is a D,D-carboxypeptidase that depletes the D-ala precursor thus favoring formation of D-ala-D-lac. VanZ performs an unknown function but may be more necessary for teicoplanin resistance than vancomycin resistance.^6^

The *vanB* gene cluster is the second most common and clinically significant mechanism mediating vancomycin resistance in *E faecium*. The *vanB* gene cluster typically leads to vancomycin MIC values near the vancomycin breakpoint which may result in resistance. Like *vanA*, *vanB* gene products alter the peptidoglycan terminal sequence from D-ala-D-ala to D-ala-D-lac.^5,7^ Although some of the genes are analogous to those of *vanA*, the *vanB* cluster primarily differs in its regulatory genes (*vanR*_*B,*_ *vanS*_*B*_ *)*. This difference is thought to allow vancomycin to induce expression of the *vanB* gene cluster, but not teicoplanin.^7^

## Genotypic mechanisms associated with VVE and reversion to vancomycin resistance

A variety of mechanisms may lead to the genotypic-phenotypic mismatch found in VVE as well as the subsequent reversion to vancomycin resistance. First, several studies report frameshifts or complete or partial deletions of *vanRS*, inhibiting the organism’s ability to detect vancomycin and therefore promote *vanHAX* transcription.^4,8–16^ In these reports, transition from phenotypic vancomycin susceptibility to resistance was mediated by mutations that allowed *van*H to utilize other promoter mechanisms instead of *vanRS.* In one example, *vanHAX* was incorporated into the chromosomal DNA of the organism allowing use of a constitutively active ribosomal RNA gene promoter.^14^ Others report mutations in the *vanH* promoter itself that allow for constitutive activity, obviating the need for activation by vanRS.^10^ Second, another set of reports describe deletions in *vanX* leading to the genotypic-phenotypic mismatch in VVE.^13,15,17^ These deletions result in an enzyme less proficient in cleaving D-ala-D-ala, thus allowing the isolate to retain susceptibility to vancomycin. Transition from phenotypic vancomycin susceptibility to resistance is mediated by alternative mechanisms to decrease the amount of D-ala-D-ala available, favoring formation of D-ala-D-lac. One such mechanism is an increase in *vanA* plasmid copy number to produce more VanX enzyme. Another includes mutations that inactivate ddl, an enterococcal ligase outside of the *vanA* operon that forms D-ala-D-ala.^18^ Finally, one report^1^ describes silencing of *vanHAX* expression via an upstream insertion sequence, *ISL3*, as a mechanism to retain vancomycin susceptibility. Excision of *ISL3* in the presence of vancomycin then accounted for the transition from phenotypic vancomycin susceptibility to resistance^2^.

VVE has also been described amongst *E. faecium* isolates with *vanB* genotype. Hashimoto *et al.* reported single amino acid substitutions in the *vanB* cluster were associated with vancomycin susceptibility and demonstrated reversion to phenotypic vancomycin resistance upon vancomycin exposure.^7^ The mechanism underlying reversion remains unclear but may be associated with increased transcription and thus expression of the *vanB* gene cluster and possibly mutations in the regulatory *vanS*_B_ gene^19^ or other mutations outside of the *vanB* cluster.^7^

## Microbiology of VVE

Microbiology labs may use genotypic methods to detect vancomycin resistance among enterococcal isolates in addition to routine phenotypic antimicrobial susceptibility testing modalities. One common method is a genotypic assay for *vanA* in *E. faecium*, which has excellent sensitivity and specificity and can be performed more rapidly than conventional techniques.^20^ Genotypic testing for *vanB* is also commercially available.^21^ Both the Clinical and Laboratory Standards Institute (CLSI)^22^ and the European Committee on Antimicrobial Susceptibility Testing (EUCAST)^23^ have acknowledged the potential benefits of genotypic testing, though neither organization has incorporated it into their recommendations for routine work. Because of this, individual laboratories implement different protocols detailing when and how to use genotypic testing in the work-up of enterococcal isolates.

Screening for vancomycin-resistant enterococci (VRE) may involve use of a vancomycin-impregnated chromogenic agar, which is a selective and differential media that uses color to distinguish *E. faecalis* and *E. faecium* from other enterococci with intrinsic vancomycin resistance. This method may be a preferred way of assessing VRE colonization in healthcare settings due to ease of throughput and low cost.^24^

Early VVE detection is only possible when a combination of genotypic *vanA* testing and phenotypic susceptibility testing is used. Phenotypic methods such as disk-diffusion and traditional methods for determining a Minimum Inhibitory Concentration (MIC) will not detect VVE as these isolates are phenotypically susceptible to vancomycin. Studies have reported a range in vancomycin MIC from 0.38 to 2 μg/mL with MIC of the revertant commonly >256 (Table 1). Similarly, chromogenic agar will also not detect VVE. Neither the selective component of the chromogenic agar nor the dose and duration of vancomycin used during susceptibility testing induce the reversion mutations necessary to lead to a detectable vancomycin-resistant phenotype.

**Table 1.** Description of genetic mechanisms described for *VanA*-positive vancomycin-variable *E. faecium* isolates from published manuscripts from 2004 to 2023

Reference	Year (isolates isolated)	Country	Number and source of tested isolates	Isolate/Clone	Mechanism conferring vancomycin susceptibility	Reversion mechanism restoring vancomycin resistance	Initial vancomycin MIC range in μg/mL (Reversion MIC range in μg/mL)	Daptomycin-susceptible	Linezolid susceptible
North America									
Gagnon^4^	2008–2010	Canada	6, surveillance	NR	Full deletion of *vanR* and *vanS* (5) Deletions in *vanS* (1)	NR	0.5–2 (NR)	Yes	Yes
Szakacs^8^	2009–2011	Canada	44, surveillance 8, clinical	15/20 tested identical by PFGE and MLST (strain not identified)	Insertion sequence replacing *vanRS*	NR	1 (NR)	NR	NR
Coburn^9^	NR	Canada	1, clinical	NR	Full deletion of *vanRS*	NR	1 (>256)	Yes	Yes
Thaker^10^	NR	Canada	4, clinical	NR	Insertion sequence with full deletion of *vanRS*	Insertion sequence upstream of *VanH* in promoter	<1–2 (>32–256)	NR	NR
Europe									
Sivertsen^1^	2013–2014	Norway	52, clinical and surveillance	ST203	Insertion sequence between *vanR* and *vanHAX*	Insertion sequence excision	0.75–2 (>256)	NR	NR
Hammerum^17^	2015–2019	Denmark	77, clinical	ST203 ST1421 ST30	Deletions in *vanX*	Disrupted ddl function Increased plasmid copy number	NR (NR)	NR	NR
Hansen^13^	2016–2017	Denmark	48, surveillance	ST1421	Deletions in *vanX* (48) Deletions in *vanS* (3) Frameshift of *vanS* (2) Insertion in *vans*	Increased plasmid copy numbers Inactivation of *ddl*	≤4 (256)	NR	NR
McInnes^14^	2016–2017	United Kingdom	1, surveillance	NR	Insertion sequence in *vanR* gene and promoter	Integration of *vanHAX* into chromosome with upstream ribosomal RNA promoter	1 (512)	NR	NR
Wagner^12^	NR	Sweden	1, surveillance	ST203	Deletions in *vanR*	Partial deletion in *vanHAX* promoter	1 (>256)	NR	NR
Asia									
Jung^15^	2004–2008	South Korea	18, clinical	ST78 (50%) ST192 (22%) ST17 ST18 ST320 newST All but new ST in CC17	Insertion sequence in *vanR* Insertion sequence in *vanH* promoter Full deletion of *vanH* and *vanX*	NR	0.38–2 (NR)	NR	NR
Kim^28^	2016–2018	South Korea	5, clinical	NR	Presumed to be *vanRS* mutations based upon previous papers^8,10^	NR	NR (NR)	NR	NR
Sugumar, Viswanath^16,29^	2019–2020	India	5, clinical	ST80 ST18 ST31 (all CC17)	Full deletion of *vanRS* (4) Full deletion of *vanR* (1)	NR	0.5–1 (NR)	NR	Yes
Australia									
Wagner^11^	2015–2016	Australia	8, clinical	ST1421	Deletions in *vanRS*	Partial deletion in *vanHAX* promoter	1 (8–256)	NR	NR
Merlino^29^	NR	Australia	1, clinical	ST 1424	Deletions in *vanA* or Full deletion of *vanXY*	NR	≤0.5 (NR)	Yes	NR

NR, Not reported; MIC, Minimum Inhibitory Concentration.

Surveillance typically refers to surveillance for VRE using rectal cultures, with varying specific institutional protocols.

Clinical isolates were detected from clinical specimens including blood, urine, wounds, bile, and ascites.

CLSI^22^ and EUCAST^25^ recommend that any *Enterococcus* isolate that tests positive for *vanA* or demonstrates resistance to vancomycin on routine antimicrobial susceptibility testing be reported as vancomycin-resistant. EUCAST additionally recommends reporting these isolates as teicoplanin resistant. This conservative approach accounts for the possibility of VVE as well as potential errors in conventional testing methods in the presence of discrepant results.

## Epidemiology of VVE

One of the first well-described reports of VVE was from Quebec, Canada, where six cases were detected from rectal swab samples from hospitalized patients during routine VRE surveillance.^4^ Of note, genotypic *vanA* and phenotypic susceptibility testing was routinely done on all isolates, allowing for the detection of VVE. Subsequent reports from Denmark^17^ and India^26^ confirmed that this organism is found worldwide (Table 1). Other reports describe the proportion of VVE among VRE isolates. Sazacs, et al. reported that 15% of patients from a 2-year VRE outbreak investigation in Toronto had VVE (44/285); 36% of these were identified during admission screening.^8^ A subsequent study from a network of hospitals in Toronto reported that 47% (18/38) of *vanA* positive *E.faecium* clinical isolates were VVE.^27^

Like VRE, VVE has been associated with nosocomial outbreaks. Sivertsen, et al. reported an extensive outbreak investigation after two patients with VVE of the same sequence type (ST203) were identified in Norway.^1^ Of 15,158 clinical and surveillance samples screened during an 18-month period, 93 were VVE and 1 clone dominated on pulse field gel electrophoresis, suggesting nosocomial spread. Sequencing of some of these isolates demonstrated a plasmid with a *vanA* gene cluster variant different from Tn1546 which is typically associated with *vanA* positive VRE. Importantly, the authors also detected this plasmid in one *E. faecalis* isolate, raising the possibility of horizontal inter-species transfer of mobile genetic elements. Hansen et al. reported another extensive outbreak investigation in Denmark in which the VVE clone (ST1421-CT1134) was identified and subsequently became the dominant *vanA* positive *E. faecium* clone in the country by 2019.^13,17^ This same clone has been reported in outbreaks in Australia.^11^

Given the challenges of detecting VVE, it is likely that VVE from both screening and clinical samples is underreported. Available data also suggests that once this organism becomes endemic in an institution or region it is likely to spread and the routine microbiologic approach to evaluating *vanA* positive enterococci may need to be adjusted.

## Clinical reports of VVE

Because most early clinical reports of VVE described treatment emergent vancomycin resistance, clinicians questioned if vancomycin was effective therapy for infections caused by these organisms. Coburn, et al. described a patient with *vanA* positive, vancomycin-susceptible *E. faecium* isolated from ascitic fluid who was found to have a clonal vancomycin-resistant *E. faecium* on rectal swab after 8 days of vancomycin therapy.^9^ The two patients who prompted the outbreak investigation described by Sivertsen et al. both developed clinical failure and subsequently had vancomycin-resistant *E. faecium* isolated from wound or blood cultures after approximately 7 days of vancomycin therapy.^1^ In a retrospective analysis comparing VVE to VRE and vancomycin-susceptible *E. faecium* cases, Kohler et al found no association between VVE and breakthrough bacteremia, but a majority of these cases were treated with agents active against VRE. They noted that acquisition of VRE and VVE had similar risk factors compared to patients infected with vancomycin-susceptible *E. faecium*. Those with VRE or VVE were more likely to have prior antibiotic exposure (though not exclusively vancomycin) and more likely to have a central venous catheter as a source of infection. Notably, these authors also found no association between VVE and 30-day mortality.^27^

Laboratory-based studies demonstrate reversion of VVE from a vancomycin-susceptible to a vancomycin-resistant phenotype, but it is unclear how frequently this reversion occurs within a bacterial population.^10,17^ Jung, et al. found that 22% (4/18) of VVE isolates reverted to vancomycin resistance upon laboratory exposure to either vancomycin or teicoplanin.^15^ Wagner et al, found that after 48 hours vancomycin exposure, reversion to vancomycin resistance happened frequently, yet still below the threshold of detection for standard susceptibility testing.^11^ These findings suggest vancomycin should be avoided when treating infections caused by *vanA* positive clinical isolates, regardless of phenotypic susceptibility testing, which is in line with current CLSI^22^ and EUCAST^25^ guidelines.

Although phenotypic reversion has been described in the laboratory and clinical setting, further study is needed to determine which factors affect this phenomenon including site of infection and degree of vancomycin exposure (eg, duration and drug levels). Additionally, as many hospitalized patients receive vancomycin for other clinical reasons, we do not know how or if vancomycin exposure prior to clinical infection may further affect observed resistance patterns.

## Conclusion

In this review, we have described known epidemiologic, microbiologic, and clinical aspects of VVE. However, much remains to be learned about this variant of enterococcus. In the interim, clinicians should be aware of existence of and local prevalence of VVE, understand the potential limitations in their institution’s microbiologic approach to evaluating *E. faecium* isolates, and how this may affect clinical management.
